# Bioanalytical Validated Spectrofluorimetric Method for the Determination of Prucalopride succinate in Human Urine Samples and Its Greenness Evaluation

**DOI:** 10.1007/s10895-023-03150-1

**Published:** 2023-02-17

**Authors:** Marwa T. Saad, Hala E. Zaazaa, Taghreed A. Fattah, Shereen A. Boltia

**Affiliations:** 1 Pharmaceutical Chemistry Department, National Organization for Drug Control and Research (NODCAR), Giza, Egypt; 2 Analytical Chemistry Department, Faculty of Pharmacy, Cairo University, Qasr El-Aini St., Cairo, 11562 Egypt

**Keywords:** Prucalopride Succinate, Spectrofluorimetry, Urine, EMA Bioanalytical Validation

## Abstract

An economical & eco-friendly spectrofluorometric method has been developed for the determination of prucalopride succinate (PRU) in human urine on the basis of the drug’s native fluorescence. The type of solvent and the wavelengths of excitation and emission have been carefully selected for optimal experimental conditions. In deionized water, the fluorescence intensity was measured at λ emission 362 nm upon excitation at 310 nm. This bio-validated method was carried out using 30uL urine without any preliminary steps. The calibration curve for prucalopride succinate shows a linear relationship in a concentration range of 0.75–5.5 µg/mL. Accuracy and precision were obtained using 4 quality control samples which are: 0.75 μg/ mL (LLOQ), 2.25 μg/mL (QCL), 2.5 μg/mL (QCM) & 4.125 µg/mL (QCH). The validation of this proposed technique obeys European Medicines Agency (EMA) Guidelines for validating bioanalytical methods and the greenness assessment was evaluated according to the Analytical GAPI approach.

## Introduction

Bioanalysis has a great concern about identifying and quantifying many drugs in different biological matrices like tissue, saliva, blood, urine, and plasma. Validation of any analytical procedure has critical importance in acquiring well-grounded findings. In the case of bio validation, it helps in covering toxicological studies, pharmacokinetic steps & items related to sample preparation, storage, transportation, handling, and collection [1]. Reliable measurements have great importance to make satisfactory decisions for accurate drug dosing and monitoring patient safety. So, it is an aspect of critical importance.

**Fig. 1:**
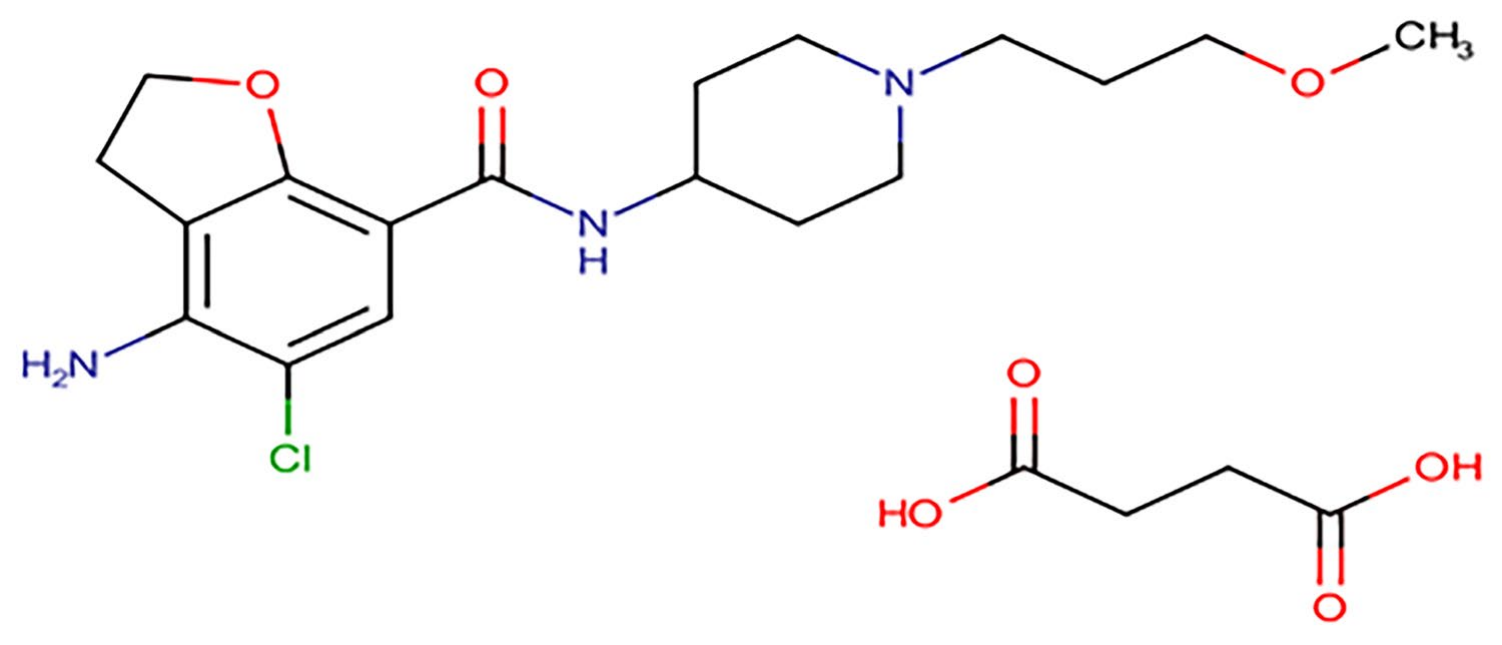
Chemical structure of prucalopride succinate

Prucalopride succinate [2] (PRU) Fig. 1 is chemically known as 4-amino-5-chloro-*N*-[1-(3-methoxypropyl) piperidin-4-yl]-2,3-dihydro-1-benzofuran-7-carboxamide; butanedioic acid used as prokinetic agent to relieve constipation. It is formulated as Resolor® tablets.

Dosage adjusted regimens are important for both normal and renal impaired patients. As renal impaired patients have a reduction in their drug excretion capability that has been principally excreted in urine resulting in toxicity& life-threatening if the dose of the drug not adjusted in a proper way. Also, urinary excretion data prevents drug-drug interactions either with synergism or antagonism. Prucalopride succinate has been reported to be excreted unchanged in urine 60–65% [3]. So, it facilitates the performance of excretion studies.

The literature review revealed the determination of PRU using various analytical techniques including chromatographic [4–10], spectrophotometric [11] methods and only one reported spectrofluorometric procedure that uses luminescence reaction [12] but there is no quantification in the urine sample. Spectrofluorimetric technique is commonly used in drugs analysis either in dosage form or in biological fluids for its high sensitivity and simplicity [13–15]. By taking the benefit of native fluorescence of PRU; the proposed method is aimed at developing an easy and rapid assay method for the quantitative determination of PRU in human urine without any time-consuming sample preparation steps for routine analysis. Also, it could be useful for the biopharmaceutical characterization of drug products (monitoring of the levels of PRU in urine in bioavailability testing, for the evaluation of in vitro–in vivo correlation and screening of different formulations during drug product development).

## Experimental

### Instrument

Agilent Cary Eclipse Fluorescence Spectrofluorimeter equipped with Agilent Xenon flash lamp and 10-mm matched pair of stoppered quartz cells. Operating of this system done via Cary Eclipse WinFLR software.

### Materials and Reagents

#### Standard Samples

PRU working standard was obtained from Mash Pharmaceutical Industries Cairo, Egypt. Its purity was certified to be 99.5%.

#### Pharmaceutical Dosage Form

Resolor ® tablets their batch number (JGL5A00), each single dose containing 2 mg of PRU. Manufacturing process took place by Janssen-Cilag and available in Egyptian market.

### Reagents & Chemicals

Sodium hydroxide, sulphuric acid (Piochem, Egypt). HPLC grade methanol, acetonitrile and ethanol (Fisher Chemical, UK).Urine sample has been collected from a female volunteer in good health. Deionized water was acquired from an Elga Ultrapure Q apparatus.

### Preparing of Standard Solutions

Stock solution of 0.5 mg/mL PRU has been prepared using deionized water. This solution was further diluted with the same solvent to get the working solution (25 µg/mL) to construct the calibration curve with the aid of calibration points which were 0.75, 1.25, 1.75, 2, 3.25, 3.5, 5.5 μg/ mL; and quality control samples (QCs) as: 0.75 μg/ mL (LLOQ), 2.25 μg/mL (QCL), 2.5 μg/mL (QCM) & 4.125 µg/mL (QCH).

### Preparing of Dosage Form

A weight of through roughly crushed powder of 7 Resolor ® tablets equivalent to 12.5 mg of PRU has been transferred into a 25-mL volumetric flask, subsequently, dilution with 25-mL Deionized water and stirring for 30 min by magnetic stirrer took place for reaching the final concentration of 0.5 mg/mL. The drug solution has been filtrated through a syringe filter, then 1.25 mL of the filtrated PRU solution was transferred into 25-mL volumetric flask rendering a concentration of (25 µg/mL) from which 0.8 mL withdrawn into 10-mL volumetric flask with addition of 30uL urine, then the volume was completed with deionized water to get a concentration of 2 µg/mL. The FI was measured and concentration was calculated from the calibration curve.

### Assay Procedure

#### Final Sample Preparation

Different aliquots of PRU were added to 30µL of centrifuged human urine in 10 mL volumetric flasks then the volume of each flask was completed to the mark with deionized water to acquire final relevant concentrations of 0.75, 1.25, 1.75, 2, 3.25, 3.5, 5.5 μg/mL PRU. QC samples (0.75, 1.5, 2.5, and 4.125 μg/mL) were also formulated in the same manner. The fluorescence intensity (FI) was measured at emission 362 nm after the excitation step at 310 nm versus blank prepared in an identical way. Constructing of calibration curve was accomplished by using recorded FIs & the related concentration of PRU plotted against each other, then regression equation was calculated.

### Analysis of PRU in Spiked Urine Samples

Different concentrations of PRU ranges between (0.75–5.5 µg/mL) were monitored after the addition of 30µL urine derived from a healthy female volunteer (20 years, 80 kg, 170 cm height). Before& during carrying out the study the volunteer was informed to cease any remedies for a couple of weeks. Urine samples were centrifuged ten minutes before usage. Centrifuged 30µL of urine & different concentrations of PRU were added together in 10 -mL volumetric flasks then the volume was completed to the mark with deionized water. The FI was estimated versus blank prepared in a similar way.

### Bio-Analytical Method Validation

This designed procedure follows EMA Guidelines for validation of Bioanalytical Methods [16].

#### Construction of Calibration Curve

Six calibration curves have been accomplished on consecutive days by plotting the resulted FI values against respective concentrations of PRU in urine in the range from 0.75 to 5.5 μg/mL.

#### Estimation of Lower Limit Of Quantification (LLOQ)

LLOQ is considered the lowest calibration standard (0.75 μg/mL PRU), it was estimated 5 times followed by the determination of accuracy and precision in terms of % recovery and %RSD, respectively.

#### Evaluation of PRU Stability

Three determinations of QCL& QCH were measured after exposure to various storage conditions. The QC samples were analyzed against a calibration curve of the day and the acquired concentrations were compared to the nominal concentrations. The mean concentration at each level should be within ± 15% of the nominal concentration.

##### Short term stability

FI of three determinations was recorded using low and high QC samples that were kept at room temperature for 24 h then the results were compared against the calibration curve of freshly spiked calibration standards.

##### Freeze and Thaw Stability

FI of three determinations was recorded using low & high QC samples that were exposed to three freeze–thaw cycles of − 80° C in three consecutive days and comparing results against a calibration curve of freshly spiked calibration standards.

##### Long-Term Stability

FI of three determinations was recorded using low &high QC samples that have been frozen in the freezer (− 80° C) for 30 days. The results have been deduced from the calibration curve of the day. The % CV must be within ± 15% of the nominal concentration.

## Results and Discussion

Bioanalytical validation could be performed according to EMA or FDA Guidance. Both documents have similarities but are not identical. For example, the acceptance criteria for accuracy have been reported in EMA Guidance to the mean concentrations, but it is unclear if the FDA’s criteria are related to the mean or to each sample. Only the EMA specifies how to study the matrix effect not only recommendations like FDA. EMA Guidance shows in a more precise way the practical conduct of experiments, while the FDA shows reporting recommendations in a more comprehensive way. Among the benefits of using EMA guidance is that found to have more flexibility regarding whether the information is established in set in SOPs or study plans and supply the ability to laboratories having GLP certification to construct quality systems fitting their own purpose. The EMA supply a consistent presentation of numerical values by using accurate formulas such as calculating accuracy and precision. [1]

Generally, using biological specimens in performing laboratory drug testing can provide various levels of sensitivity, specificity and accuracy. Urine is mostly preferred biological specimen as it is easy collected also high concentrations of drugs & metabolites can be found in urine which permits longer detection times than concentrations in serum [17].

In vitro tests are performed in the laboratory, and it involves the study of microorganisms, human or animal cells in culture. This methodology helps in evaluating different biological phenomena in certain cells without the distractions and potential confounding variables present in whole organisms.

Researchers can conduct more detailed analyses and investigate biological effects in a larger number of in vitro subjects than they would in animal or human trials. Despite positive preclinical findings, approximately 30% of drug candidates fail human clinical trials because of unwanted effects. An additional 60% do not achieve the desired effect [18].

PRU is extensively eliminated unchanged in urine 60–65%. By taking into consideration the benefit of PRU native fluorescence besides the easiness of spectrofluorimetric procedure, a spectrofluorimetric method was validated to determine PRU in the urine of humans using a small amount of urine (30µL) and deionized water that was chosen for its best sensitivity beside its greenness character.

### Method Optimization

**Fig. 2:**
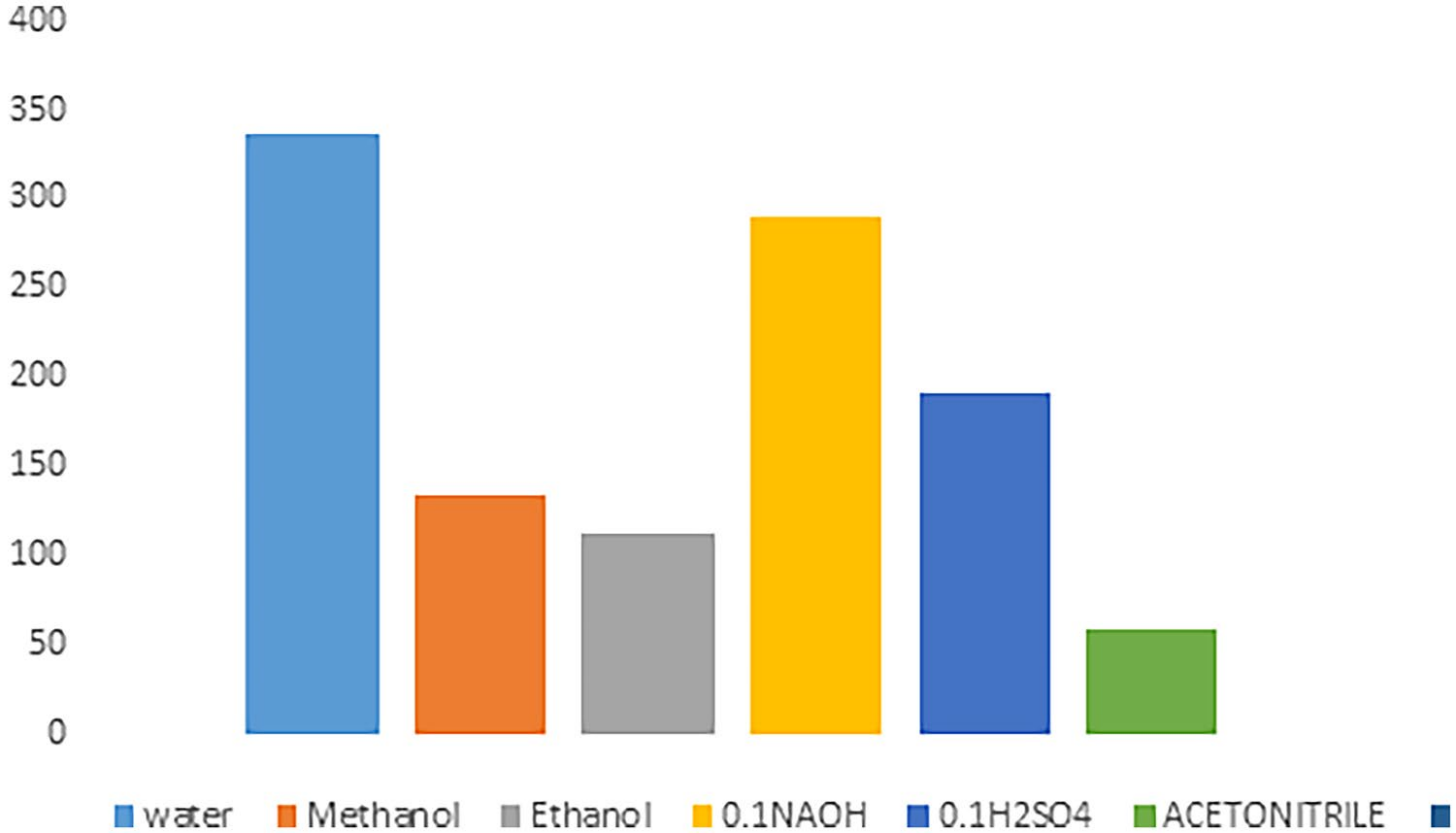
Effect of solvents

**Fig. 3:**
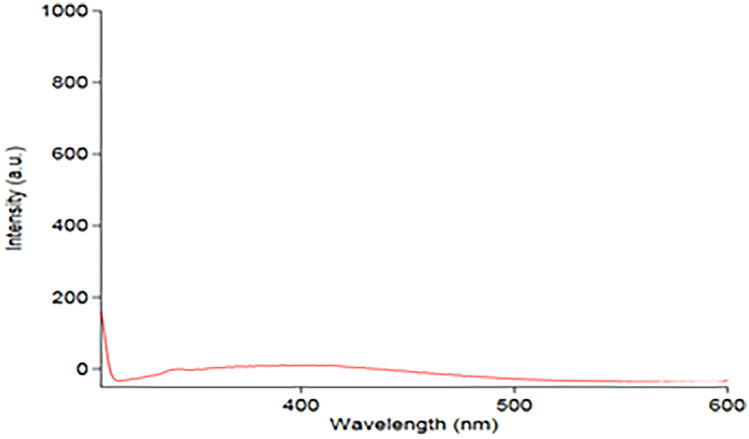
Emission spectrum of urine sample against water

**Fig. 4:**
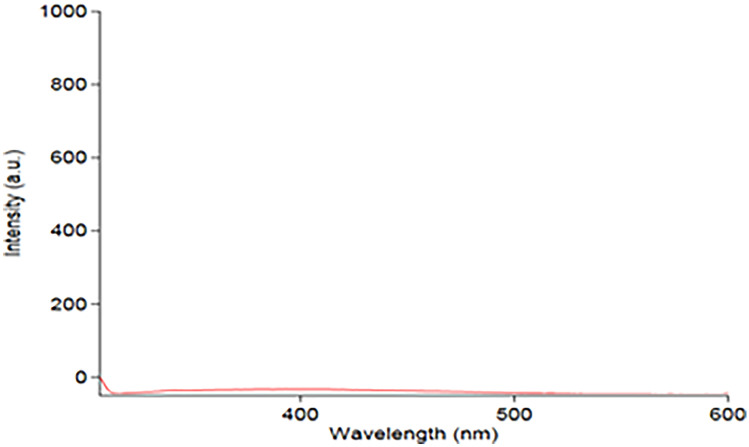
Emission spectrum of urine sample against urine

**Fig. 5:**
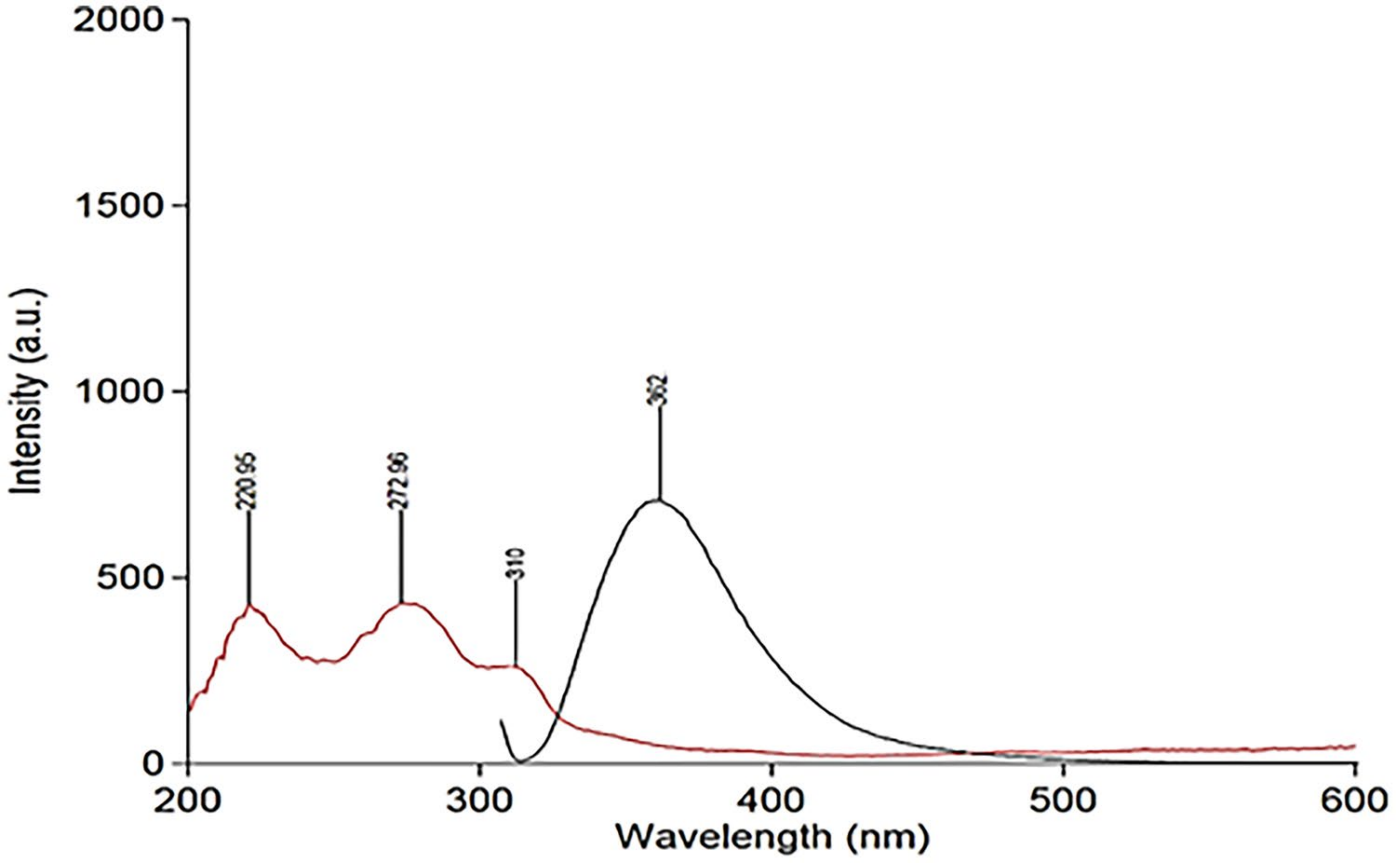
Overlay spectra of excitation and emission at λ excitation of 310 nm and λ emission 362 nm

The results of studying the effectiveness of various diluting solvents were recorded. Figure 2 illustrates that the deionized water provides the greatest FI value. The selection of wavelengths of both excitation & emission wavelengths was tested depending on the ability of the used instrument to scan PRU excitation & emission wavelengths. PRU has 3 excitation wavelengths at 220, 272& 310 nm. The latter was chosen as the best excitation wavelength although its low sensitivity because it gives the least blank reading, Figs. 3 and 4. While 362 nm was found to be the optimal emission wavelength in terms of sensitivity as shown in Fig. 5.

### Validation Procedures

Bioanalysis is concerned with identifying and quantifying analytes in different biological matrices. Validation aids in the achievement of dependable results which are demanded for proper drug dosing and keeping patient safety. Validation of this designed analytical procedure obeys the EMA Guidelines for Bioanalytical Method Validation [16].

#### Selectivity

Six individual sources of blanks of urine matrix that individually analyzed to exclude any interfering components excreted with PRU and there is no interference is recorded.

#### Plotting Calibration Curve

Six calibration curves have been constructed using various concentrations ranging from0.75 to 5.5 μg/mL PRU providing linear relationship and excellent recoveries (Table 1).

**Table 1:** Data obtained from the different calibration curves

Calibration number	Slope	Intercept	r
1	113.47	101.64	0.9996
2	112.89	97.128	0.9996
3	111.56	85.705	0.9993
4	96.28	106.85	0.9995
5	111.29	91.121	0.9999
6	110.33	98	0.9995

^*^Mean of slope = 109.30

^*^Mean of intercept = 96.74

^*^7 points were used in each calibration curve

#### Lower Limit Of Quantification (LLOQ)

The analyte signal of the LLOQ sample should be at least 5 times the signal of a blank sample as shown in Fig. 3a and b.

The lower limit of quantification (LLOQ) is found to be 0.75 μg/mL. The %CV was calculated. And found to be 1.49, and it is considered an accepted value.

#### Accuracy & Precision

Within and between run accuracy and precision for PRU were estimated by using QC samples as illustrated in (Table 2) giving accepted values.

**Table 2:** Results of accuracy and precision for determination of PRU in human urine

	Within-run	Between-run
LLOQ	99.36 ± 7.38	99.85 ± 9.87
QCL	104.21 ± 7.27	102.13 ± 9.00
QCM	99.73 ± 1.99	101.17 ± 4.73
QCH	100.44 ± 2.51	98.84 ± 5.33
n	5	5

#### Recovery

This technique was carried out directly without extraction thus recovery is supposed to be around 100%. The presence of urine suppressed the FI, results were consistent in all the QC samples. Although the matrix effect of urine was high in suppressing PRU native fluorescence (around 15%) but the FI is still high to give the required sensitivity (Table 3).

**Table 3:** Matrix effect of PRU

PRU	FI value (PRU with urine)	FI value (PRU only)	Recovery % (FI PRU _with urine/ without urine_ ∗ 100)
QCL (1.5ug)	291	340	85.58
	287	339	84.66
	288	340	84.70
	290	339	85.54
	288	338	85.20
	287	338	84.91
Mean ± SD	288.5 ± 1.64	339 ± 0.89	85.10 ± 0.3
CV%	0.56	0.26	0.37
QCH (4.125 µg)	525	581	90.36
	521	585	89.05
	523	580	90.17
	525	581	90.36
	521	583	89.36
	523	584	89.55
Mean ± SD	523 ± 1.78	582.33 ± 1.96	89.76 ± 0.52
CV%	0.34	0.33	0.58

#### Evaluation of Stability

Short-term stability: FI of three determinations were recorded using low and high QC samples that were kept at room temperature for 24 h.

Freeze and thaw stability: The stability of PRU after three freeze–thaw cycles was determined by recording FI of three determinations using low & high QC samples that were exposed to three freeze–thaw cycles of − 80° C in three consecutive days.

Long-term stability: it was studied where PRU in urine matrix stored in the freezer at − 80 °C for 30 days using low & high QC samples then recording FI of three determinations.

Generally, samples are considered stable as the results are within of the original concentration ± 15% of the nominal concentration. All stability samples were compared against a calibration curve of freshly spiked calibration standards. The results are discussed briefly in (Table 4).

**Table 4:** Stability data of PRU

	Mean recovery ± RSD % (*n* = 3)		
	short term stability	Freeze thaw cycle	Long term stability
QCL	103.51 ± 5.79	94.77 ± 3.59	92.65. ± 6.53
QCH	99.58 ± 1.78	98.21 ± 3.26	95.28 ± 3.70

#### Analysis of PRU in Dosage Form & Evaluation of Standard Addition Technique

The proposed method was applied for the analysis of PRU dosage form after extraction and spiking in human urine and good recoveries were obtained as shown in Table 5. Also to assess the validity of the method on dosage form, application of standard addition technique was performed using 1, 1.25, 2, 2.5 µg of standard PRU, where good recoveries were obtained as shown in Table 6.

**Table 5:** Application of the proposed method for the determination of PRU in a pharmaceutical dosage form and application of the standard addition technique

Preparation	Drug/Claimed potency	Claimed taken	%Found
Resolor® tablets	PRU/ 2 mg per tablet	2 µg/mL	94.65 ± 1.47
B.N. JGL5A00			

^*^Average of three determinations

**Table 6:** Application of the standard addition technique

Standard Addition Technique	
Pure Added (µg/mL)	%Recovery (Mean ± SD)
1	100.41 ± 0.30
1.25	100.56 ± 1.00
2	98.48 ± 0.58
2.5	98.11 ± 0.83

^*^Average of three determinations

### Greenness Evaluation of the Recommended Spectrofluorimetric Method

**Fig. 6:**
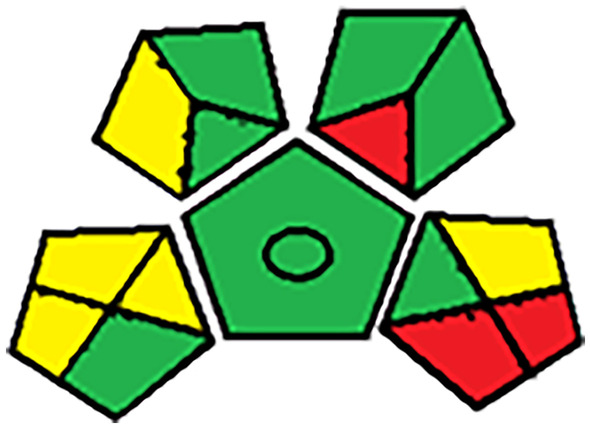
Greenness assessment using GAPI approach

Analytical GAPI approach [19] is utilized to evaluate& quantify various environmental factors involved in steps of analytical methodology. Plotka-Wasylka presented the green analytical procedure index (GAPI) which acts as a combination of benefits of both the NEMI and Eco-scale. Also, it can evaluate the green character in the suggested method. GAPI can assess any analytical procedure using 15 factors, beginning with the sample preparation, reagents and solvents, instrumentation, waste and waste treatment. As shown in Table 7, all the assessed parameters have been represented in a colorful picogram based on their environmental impact that is either green, yellow or red according to the table listed in the GAPI approach Fig. 6.

**Table 7:** Greenness assessment using GAPI approach

Category	Green	Yellow	Red
Sample preparation			
Collection (1)	In-line	On-line or at-line	Off-line
Preservation (2)	None	Chemical or physical	Physico-chemical
Transport (3)	None	Required	-
Storage (4)	None	Under normal conditions	Under special conditions
Type of method: direct or indirect (5)	No sample preparation	Simple procedures, eg. filtration, decantation	Extraction required
Scale of extraction (6)	Nano-extraction	Micro-extraction	Macro-extraction
Solvents/reagents used (7)	Solvent-free methods	Green solvents/reagents used	Non-green solvents/reagents used
Additional treatments (8)	None	Simple treatments (clean up, solvent removal, etc.)	Advanced treatments (Derivatization, mineralization, etc.)
Reagent and solvents			
Amount (9)	< 10 mL (< 10 g)	10–100 mL (10–100 g)	> 100 mL (> 100 g)
Health hazard (10)	Slightly toxic, slight irritant; NFPA health hazard score = 0 or 1	Moderately toxic; could cause temporary incapacitation; NFPA = 2 or 3	Serious injury on short-term exposure; known or suspected small animal carcinogen; NFPA = 4
Safety hazard (11)	Highest NFPA flammability or instability score of 0 or 1. No special hazards	Highest NFPA flammability or instability score of 2 or 3, or a special hazard is used	Highest NFPA flammability or instability score of 4
Instrumentation			
Energy (12)	≤ 0.1 kWh per sample	≤ 1.5 kWh per sample	> 1.5 kWh per sample
Occupational hazard (13)	Hermetic sealing of analytical process	-	Emission of vapours to the atmosphere
Waste (14)	< 1 mL (< 1 g)	1–10 mL (1–10 g)	> 10 mL (< 10 g)
Waste treatment (15)	Recycling	Degradation, passivation	No treatment
ADITIONAL MARK: QUANTIFICATION			
Circle in the middle of GAPI: *Procedure for qualification and quantification*		No circle in the middle of GAPI: *Procedure only for qualification*	
NFPA: National Fire Protection Association

## Conclusion

The proposed method has been established to indicate the fluorimetric behavior of PRU using EMA guidelines for its bio validation in human urine. This method can be used for pharmacokinetics investigations as spectrofluorimetry is a rapid, reliable & low-cost technique.

We look forward to further research using the fluorescent characterization of PRU to selectively evaluate the drug in vivo for more clinical information to keep the safety of the patient & proper drug dosing.

## Data Availability

Data will be made available on request.
